# Recruitment, consent and retention of participants in randomised controlled trials: a review of trials published in the National Institute for Health Research (NIHR) Journals Library (1997–2020)

**DOI:** 10.1136/bmjopen-2021-059230

**Published:** 2022-02-14

**Authors:** Richard M Jacques, Rashida Ahmed, James Harper, Adya Ranjan, Isra Saeed, Rebecca M Simpson, Stephen J Walters

**Affiliations:** School of Health and Related Research, The University of Sheffield, Sheffield, UK

**Keywords:** statistics & research methods, audit, public health

## Abstract

**Objectives:**

To review the consent, recruitment and retention rates for randomised controlled trials (RCTs) funded by the UK’s National Institute for Health Research (NIHR) and published in the online NIHR Journals Library between January 1997 and December 2020.

**Design:**

Comprehensive review.

**Setting:**

RCTs funded by the NIHR and published in the NIHR Journals Library.

**Data extraction:**

Information relating to the trial characteristics, sample size, recruitment and retention.

**Primary and secondary outcome measures:**

The primary outcome was the recruitment rate (number of participants recruited per centre per month). Secondary outcomes were the target sample size and whether it was achieved; consent rates (percentage of eligible participants who consented and were randomised) and retention rates (percentage of randomised participants retained and assessed with valid primary outcome data).

**Results:**

This review identified 388 individual RCTs from 379 reports in the NIHR Journals Library. The final recruitment target sample size was achieved in 63% (245/388) of the RCTs. The original recruitment target was revised in 30% (118/388) of trials (downwards in 67% (79/118)). The median recruitment rate (participants per centre per month) was found to be 0.95 (IQR: 0.42–2.60); the median consent rate was 72% (IQR: 50%–88%) and the median retention rate was estimated at 88% (IQR: 80%–97%).

**Conclusions:**

There is considerable variation in the consent, recruitment and retention rates in publicly funded RCTs. Although the majority of (6 out of 10) trials in this review achieved their final target sample; 3 out of 10 trials revised their original target sample size (downwards in 7 out of 10 trials). Investigators should bear this in mind at the planning stage of their study and not be overly optimistic about their recruitment projections.

**Strengths and limitations of this study**

- This is the largest comprehensive review of recruitment, consent and retention in trials to date reporting rates for 388 single and multicentre trials published in the National Institute for Health Research (NIHR) Journals Library between January 1997 and December 2020.
- As the NIHR Journals Library intends to publish all research from Efficacy and Mechanism Evaluation, Health Services and Delivery Research, Health Technology Assessment, Programme Grants for Applied Research and Public Health Research funded projects, this study has less chance of publication bias compared with a review of other journals where publishing is more selective.
- For some trials crude recruitment rates, assuming all centres were recruiting for the same time period, were calculated, these estimates may be an underestimation of the true recruitment rate.
- The review is restricted to publicly funded trials published in the NIHR Journals Library, which may limit the generalisability of the findings.

## Introduction

Randomised controlled trials (RCTs) are the ‘gold-standard’ research design for evaluating the effectiveness of interventions in health, education and policy.^1^ Conducting an RCT requires major financial investment and substantial amounts of public funding is spent in this area each year. In 2019/2020, the National Institute for Health Research (NIHR) in England awarded over £250 million of funding to 310 research projects with a substantial proportion of this invested in RCTs.^2^

There are many practical challenges associated with conducting clinical trials. The leading reason for premature discontinuation of RCTs is poor recruitment of participants^3 4^ with accrual often taking longer or being more difficult than expected. Poor recruitment can have a number of consequences including the study being underpowered if the target sample size is not met and increased costs if an extension is required.^5^ Furthermore, discontinued RCTs are less likely to be published in medical journals^3^ which has ethical implications around research waste.^6^

There have been a number of previous studies in the UK investigating recruitment and retention in publicly funded RCTs. The earliest review, a cohort of trials funded by the Medical Research Council and NIHR Health Technology Assessment (HTA) between 1994 and 2002, reported that 31% (38/122) of the trials successfully recruited to their original recruitment target, with 54% (65/122) of trials awarded a grant extension.^7^ There is evidence of a marginal improvement in these figures over time, with results from a cohort of 151 RCTs funded by the NIHR HTA programme between 2004 and 2016 finding that 40% (61/151) of trials successfully recruited to their original sample size, and 32% (49/151) of trials extended their recruitment.^8^ In the same study, the median recruitment rate was found to be 0.92 (IQR: 0.43–2.79) participants per centre per month.

Following the publication of the review by Walters *et al*^8^ in 2017, there have been several Cochrane systematic reviews looking at strategies for improving recruitment^9^ and retention^10^ of participants in RCTs. Two strategies for improving recruitment were identified with high-certainty evidence: using open trials rather than blinded, placebo controlled trials, and telephone reminders to people who did not respond to postal invitations. There has also been a systematic review of statistical models for predicting recruitment at the design stage of a clinical trial^11^ but a survey of statisticians in UK and European clinical trial networks found that 90% (62/69) did not use statistical models for recruitment prediction.^12^ In 2014, a trials methodology research priority setting exercise was conducted using a Delphi survey of directors of UK Clinical Research Collaboration registered Clinical Trials Units (CTUs).^13^ Two of the three highest priority areas were ‘Research methods to boost recruitment in trials’ and ‘Methods to minimise attrition’.

The Consolidated Standards of Reporting Trials (CONSORT) statement was first published in 1996,^14^ and revised in 2001^15^ and 2010.^16^ It is a checklist of standards for reporting how a trial was designed, analysed and interpreted, and it has been endorsed both by prominent general medical journals and many specialist medical journals.^17^ However, reporting guidelines such as CONSORT are not adopted and adhered to as much as they should be^18^ with the previous review of recruitment and retention in RCTs by Walters *et al*^8^ finding that 63% (95/151) of trials demonstrated complete compliance with CONSORT statement and reported each of the number: screened, eligible, declined consent, recruited and assessed for their primary outcome.

This review aims to update previous research on how well recruitment and retention figures are reported, and the rates of recruitment and retention in trials published in the NIHR HTA Journal between January 2004 and April 2016.^8^ In this study, we update and extend this review to look at trials published in the NIHR Journals library from January 1997 to December 2020.

## Methods

### Trial identification

Reports of individually RCTs published in the NIHR Journals Library from January 1997 to December 2020 were reviewed. Established in 2006, the NIHR is now the largest funder of health and social research in England.^2^ The NIHR Journals Library publishes five peer reviewed journals reporting the results from a range of health research areas: Efficacy and Mechanism Evaluation (EME), Health Services and Delivery Research (HS&DR), HTA, Programme Grants for Applied Research (PGfAR) and Public Health Research (PHR) (https://www.journalslibrary.nihr.ac.uk/journals/). The first volume of the HTA journal was published in 1997 whereas the other four journals are more recent with the first volumes of the HS&DR, PGfAR and PHR journals published in 2013 and the first volume of the EME journal published in 2014. Trial reports published in the NIHR Journals Library were chosen as they provide a detailed description of the research methods and study results including recruitment and retention information.

The reports for review were obtained from the NIHR Journals Library website (https://www.journalslibrary.nihr.ac.uk/—last accessed 10 November 2021) along with any published trial paper, protocol paper or trial protocol. The published International Standardised Randomised Controlled Trial Number (ISRCTN) was used where available to check the ISRCTN register of clinical trials for additional information (https://www.isrctn.com/). The titles and abstracts of all reports published in the five NIHR journals from 1 January 1997 to 31

December 2020 were checked for relevance.

### Inclusion/exclusion criteria

To ensure consistency the eligibility criteria used by Walters *et al*^8^ was adopted. Reports included in the review were of single or multicentre RCTs that were either fully or partially randomised and where recruitment to the trial had finished. Reports of trials that terminated early, either prior to completion of recruitment or following recruitment but prior to completion of follow-up were retained. Reports of two or more parallel RCTs were included as were nested parallel trials as part of another RCT. Some reports in the PGfAR journal included multiple independent RCTs and each of these trials were included separately. Reports of non-RCTs, cluster RCTs, adaptive designs, influenza vaccination trials, follow-on studies and ongoing RCTs that had not completed recruitment were excluded. Reports of external pilot/feasibility studies were excluded as they do not contribute outcome data to the main trial and are instead often used to estimate parameters such as the number of eligible participants, willingness of participants to be randomised and follow-up rates needed for the design of the main study.^19^ Reports of internal pilot trials that either went on to contribute outcome data to a full trial or were terminated prior to the full trial because of recruitment issues were included in the review.

### Data extraction

After the NIHR reports had been selected for inclusion, information was extracted using a standardised data extraction form. For each of the included trials the following information was extracted.

- Trial characteristics, including the trial design, clinical area, type of intervention, type of control, number of arms, use of blinding of trial participant, geographical region, number of centres, any support provided by a CTU and whether there was any description of pilot or feasibility work done prior to the start of the trial.
- Sample size, recruitment and retention information, including the target and actual sample size, the overall and centre-specific recruitment period and CONSORT information on the numbers screened, consented, randomised and analysed for the primary outcome.^16^

The selection of RCTs and data extraction was conducted by a team of reviewers (RMJ, RA, JH, AR and IS). Three reviewers (RMJ, RMS and SJW) conducted quality assurance checks on 30% of the included trials after the data extraction was completed, and disagreements were discussed to achieve consensus.

### Analysis

The primary outcome for the review was the recruitment rate for each trial. This was defined as the number of participants recruited and randomised per centre per month. Where explicit dates were reported the recruitment rate was calculated as the time between the date of recruitment start and the date of recruitment completion. In cases where only the months of recruitment were reported the recruitment period was estimated as the time between the first of the month and the end of the final month. If the date of the first participant recruited was reported instead of the start date of recruitment then the start of recruitment was taken as the first of the month of the first participant recruited. When the start of recruitment was not reported the start of screening was used to calculate the recruitment period. The recruitment period was estimated by subtracting the length of the follow-up period from the length of the study period when explicit information on the start and end of recruitment was not reported.

The recruitment rate was calculated in two different ways. The overall recruitment rate was calculated as the total number of participants recruited divided by the maximum number of recruiting sites, then divided by the total number of months that the trial recruited for. This overall recruitment rate is likely to be an underestimate for multicentre trials because each trial site is unlikely to open for recruitment at the same time and will not recruit for the entire recruitment period. To allow for the difference in start-up times and recruitment periods between sites, where available, the site-specific recruitment periods were extracted. These were averaged over the number of sites to give an average site-specific recruitment period. The average recruitment rate was calculated as the total number of participants recruited divided by the maximum number of sites, then divided by the average number of months that the trial recruited for.

The secondary outcomes for the review were the target sample size and whether it was achieved, the consent rate and the retention rate. The consent rate was calculated as the percentage of eligible participants that consented and were randomised (ie, the total number of participants recruited and randomised divided by the number of eligible participants). The retention rate was calculated as the percentage of randomised participants that were assessed for the primary outcome and included in the analysis of the primary outcome (ie, the number of participants included in the analysis of the primary outcome divided by the number of participants recruited and randomised).

Recruitment rates were summarised using the median and IQR due to the skewed distribution of the data.^20^ The median and IQR were also used to summarise the secondary outcomes of the consent and retention rates. Comparisons of recruitment and retention rates were made between different trial characteristics using appropriate non-parametric tests; Mann-Whitney U test (for characteristics with two levels), Kruskal-Wallis test (three or more nominal levels) and Jonckheere-Terpstra test (three or more ordered levels). Analysis was conducted on a complete case basis so where the characteristics information, recruitment rate or retention rate were missing these were excluded. All statistical analyses were conducted in R V.4.1.0,^21^ figures were produced using the package ggplot2^22^ and the Jonckheere-Terpstra test conducted using the package clinfun.^23^

### Patients and public involvement

Patients and/or the public were not involved in the design, conduct, reporting or dissemination plans of this research.

## Results

**Figure 1:**
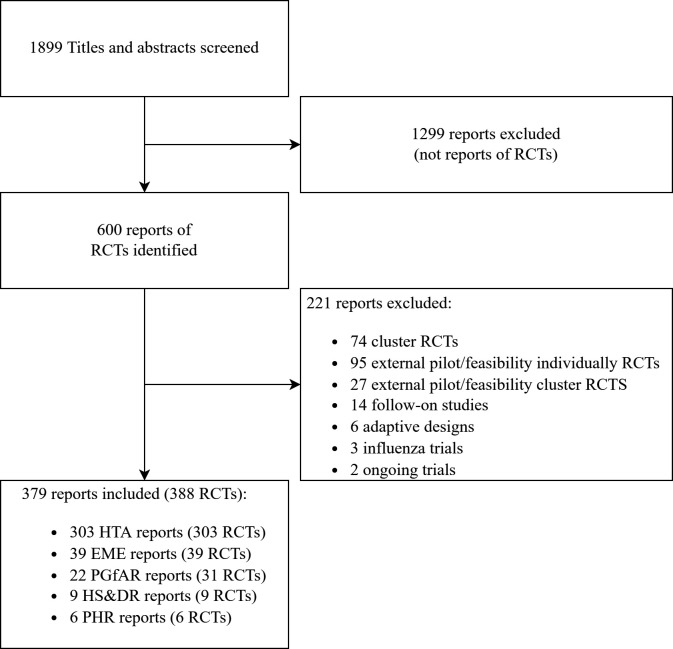
Flow diagram of search and selection process of individually RCTs from the five NIHR journals between 1 January 1997 and 31 December 2020. EME, Efficacy and Mechanism Evaluation; HS&DR, Health Services and Delivery Research; HTA, Health Technology Assessment; NIHR, National Institute for Health Research; PGfAR, Programme Grants for Applied Research; PHR, Public Health Research; RCTs, randomised controlled trials.

Between 1 January 1997 and 31 December 2020, 1899 reports were published in the five NIHR journals. Following screening, 1299 of these were excluded as reports of non-RCTs. The search identified 600 reports of RCTs of which 221 were excluded after applying the exclusion criteria (101 cluster RCTs; 95 pilot/feasibility RCTs; 14 follow-on studies; 6 adaptive designs; 3 influenza vaccination trials and 2 ongoing trials). Eight NIHR reports described the results of multiple independent trials (7 reports described 2 RCTs and 1 described 3 RCTS), therefore in total, 388 individual RCTs from 379 reports were included in the review and analysed as shown in figure 1. This includes 151 RCTs from the review by Walters *et al*.^8^

### Trial characteristics

The characteristics of the 388 trials included in the review are summarised in table 1. The most common design was a two arm parallel group, multicentre RCT. The most frequently studied clinical areas were mental health, including psychiatry and psychology (19% (73/388) of trials) and musculoskeletal conditions, including orthopaedics, rheumatology and back pain (11% (44/388) of trials). The majority of trials were set in hospitals (56% (219/388)), took place in the UK (91% (355/388)) and across multiple geographical regions (82% (317/388)). Trials of pharmaceutical interventions (29% (111/388))) were more common than other interventions and 78% (301/388) of trials used an active control. Half of all trial reports (194/388) reported or mentioned work from a pilot or feasibility study.

**Table 1:** Characteristics of the trials included in the review

Characteristic		n (%)
Trial design (n=388)	Parallel	345 (89)
	Factorial	19 (5)
	Crossover	4 (1)
	Other*	20 (5)
Arms (n=388)	2	290 (75)
	3	61 (16)
	4	24 (6)
	>4	13 (3)
Clinical area (n=388)	Mental health	73 (19)
	Musculoskeletal, orthopaedics and rheumatology	44 (11)
	Obstetrics and gynaecology	32 (8)
	Respiratory	29 (7)
	Cardiovascular	24 (6)
	Cancer/oncology	21 (5)
	Stroke	19 (5)
	Dermatology (including ulcers)	17 (4)
	Gastrointestinal	14 (4)
	Primary care	11 (3)
	Diabetes	11 (3)
	Urology	10 (3)
	Neurology	10 (3)
	Infectious disease	8 (2)
	Dentistry	5 (1)
	Other†	60 (15)
Setting (n=388)	Hospital	219 (56)
	General practice	55 (14)
	Mixed	61 (16)
	Community	34 (9)
	Other‡	19 (5)
Intervention type (n=388)	Pharmaceutical intervention	111 (29)
	Complex intervention	65 (17)
	Therapy	54 (14)
	Surgery	46 (12)
	Other§	112 (29)
Control type (n=388)	Placebo	87 (22)
	Active	301 (78)
Patient blinded (n=384)	Yes	100 (26)
	No	284 (74)
Centres outside the UK? (n=388)	Yes	33 (9)
	No	355 (91)
Geographical spread (n=388)	Multiple regions	317 (82)
	Regional	71 (18)
Some form of pilot?¶ (n=388)	Yes	194 (50)
	No	194 (50)

*Two or three parallel RCTs, cohort multiple RCT, patient preference/Zelen’s.

†Alcohol abuse, allergy, chronic fatigue, cystic fibrosis, gerontology, hepatology, intensive care, minor surgery, multiple sclerosis, obesity/weight loss, nephrology, neurosurgery, nutrition, ophthalmology, otorhinolaryngology, paediatric (general, anaesthesiology, dermatology, nephrology, obesity/weight loss), physical exercise, rehabilitation, reproductive health resuscitation, septic shock, sleep disorders, speech therapy, vascular.

‡Bowel Cancer Screening Programme, Exercise Schemes, Football Clubs, HIV Clinics, Intellectual Disability Services, Leisure Centres, Mobile Dental Clinics, Online, Physical Therapy Classes, Prison, Public School, Sexual Health Clinics, Specialist Care Centres, Stop Smoking Services, University Clinics.

§Advice and Information, consultation, diagnostic Information, drug versus surgery, equipment, health professional, patient pathway, technique.

¶Any mention of pilot work or feasibility study recorded.

RCTs, randomised controlled trials.

The recruitment and sample size characteristics of the RCTs included in the review are summarised in table 2. The majority of trials (354/388) were multicentre with a median of 17 centres (IQR: 7–37). The final recruitment target (sample size) ranged from 44 participants to 46 000 participants and the final number recruited ranged from 2 participants to 47 062. The RCT with the highest final recruitment target and highest number recruited was an obstetrics trial investigating computerised interpretation of fetal heart rate during labour.^24^ There were four trials that recruited less than 10 participants, two were discontinued at the end of an internal pilot phase due to low recruitment^25 26^ and the remaining two had no pilot phase.^27 28^ Overall, 63% (245/388) of trials recruited to their final recruitment target but 32% (79/245) of these trials required an extension to their recruitment period to meet the target. A further 22% (86/388) of trials recruited to within 80% of their final recruitment target with 36% (31/86) of these trials having an extension to their recruitment period. The original recruitment target was revised in 30% (118/388) of trials (downwards in 67% (79/118)). For the majority of trials the primary outcome was collected at between 1 and 18 months postrandomisation.

**Table 2:** Recruitment and sample size characteristics of the trials included in the review

Characteristic (n=388)		n (%)	Mean (SD)	Median (IQR)	Range
No of centres	1	25 (6)	29 (34)	17 (7–37)	1–274
	2–5	61 (16)			
	6–10	48 (12)			
	11–20	69 (18)			
	21–50	112 (29)			
	51–100	48 (12)			
	>100	16 (4)			
	Missing	9 (2)			
Original target recruitment	≤200	49 (13)	1097 (3080)	500 (300–900)	50–46 000
	201–400	101 (26)			
	401–600	86 (22)			
	601–800	41 (11)			
	>800	109 (28)			
	Missing	2 (1)			
Final target recruitment	≤200	53 (14)	1041 (3074)	480 (270–802)	44–46 000
	201–400	112 (29)			
	401–600	84 (22)			
	601–800	42 (11)			
	>800	97 (25)			
Final total recruitment	≤200	72 (19)	991 (3025)	452 (236–800)	2–47 062
	201–400	99 (26)			
	401–600	82 (21)			
	601–800	39 (10)			
	>800	96 (25)			
Final recruitment target achieved	Yes	245 (63)			
	No, but with ≥80% of target	86 (22)			
	No, <80% of target	57 (15)			
Timing of primary outcome follow-up (months postrandomisation)	≤1 month	42 (11)	12 (13)	10 (3–12)	0–120
	1< months ≤6	129 (33)			
	6< months ≤18	131 (34)			
	>18 months	63 (16)			
	Missing	23 (6)			
Timing of final follow-up (months postrandomisation)	≤1 month	20 (5)	16 (19)	12 (6–18)	0.066–144
	1< months ≤6	87 (22)			
	6< months ≤18	181 (47)			
	>18 months	88 (23)			
	Missing	12 (3)			

### CONSORT and recruitment data

Summaries of the data completeness in relation to the CONSORT statement, recruitment and retention are presented in table 3. Of the 388 RCTs identified, 68% (265/388) fully complied with the CONSORT statement and reported the number of participants screened, eligible, declined consent, recruited and assessed for the primary outcome. The total number of participants recruited and randomised, and the number included in the analysis of the primary outcome, used to measure retention, was available for all 388 trials. Regarding the information required to calculate the recruitment rate, 98% (379/388) of trials reported the number of centres, 95% (369/388) reported the maximum length of the recruitment period, and 25% (97/388) reported the centre-specific recruitment information used to calculate an average recruitment period per centre. There was enough information reported to calculate the overall recruitment rate for 94% (365/388) of trials in this review.

**Table 3:** Data completeness in relation to CONSORT guidelines and recruitment information

Trial characteristic (N=388)	n (%)
No screened	327 (84)
No eligible	309 (80)
No refused/declined consent	282 (73)
Total recruitment	388 (100)
No included in primary analysis (retention)	388 (100)
No of centres	379 (98)
Maximum recruitment length	369 (95)
Centre-specific recruitment length	97 (25)
Recruitment rate can be calculated	365 (94)

CONSORT, Consolidated Standards of Reporting Trials.

### Recruitment, consent and retention rates

**Figure 2:**
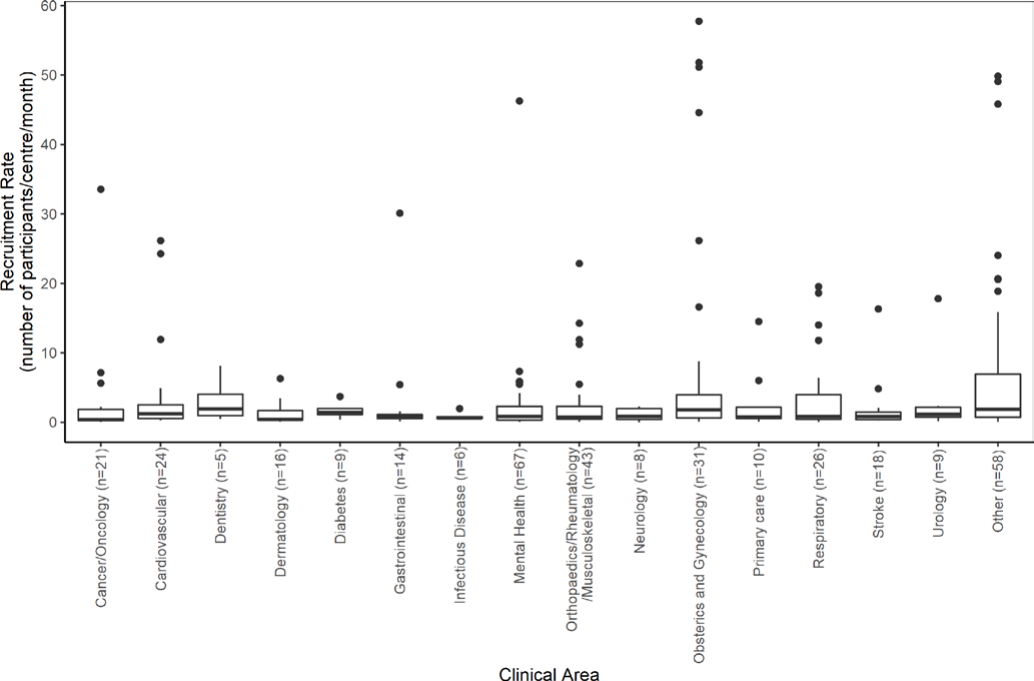
Boxplots of recruitment rates by clinical area.

From the 365 trials with sufficient information to calculate the recruitment rate, the median was found to be 0.95 participants recruited per centre per month. The highest recruitment rate (57.75 participants per centre per month) was in a trial comparing medical to surgical termination of pregnancy with a target sample size of 2232 women^29^ and the lowest (0.01 participants per centre per month) was in a trial treatment for transverse myelitis.^27^ The 80th and 90th percentiles were found to be 3.70 and 9.47 participants recruited per centre per month, respectively. From the 22 single centre trials with sufficient information, the median recruitment rate was found to be 14.12 (IQR: 4.29–26.59, range: 1.58–57.75) participants per centre per month compared with a median of 0.86 (IQR: 0.40–2.17, range: 0.01–51.14) participant per centre per month in the 343 multicentre trials. Table 4 shows some statistical evidence of a difference in recruitment rates between the five NIHR journals (p=0.010) with the PHR journal having the highest median recruitment rate (7.62, IQR: 1.79–17.06) and the HTA journal having the lowest (0.85, IQR: 0.39–2.49). However, there are only six trials from the PHR journal included in this review and three of these trials^30–32^ have a recruitment rate of 10 participants per centre per month or greater. Figure 2 shows the distribution of recruitment rates by clinical area. The highest median recruitment rate was for dentistry (1.95 participants recruited per centre per month) but this was only from five trials. The largest recruitment rates were found to be from four obstetrics and gynaecology trials,^24 29 33 34^ a mental health trial^35^ and three trials from other clinical areas.^36–38^

**Table 4:** Overall consent, recruitment and retention rates and association with Journal

	Journal	n	Median	IQR	Range	P value
Consent rate (percentage of eligible participants consented and randomised)	All	309	72%	50%–88%	4%–100%	
	HTA	230	72%	50%–88%	4%–100%	0.225*
	EME	36	74%	52%–93%	11%–100%	
	PGfAR	30	65%	48%–84%	19%–100%	
	HS&DR	7	81%	60%–97%	35%–100%	
	PHR	6	57%	40%–68%	35%–76%	
Recruitment rate (participants recruited per centre per month)	All	365	0.95	0.42–2.60	0.01–57.75	
	HTA	289	0.85	0.39–2.49	0.01–57.75	0.010*
	EME	39	1.18	0.45–2.46	0.15–18.61	
	PGfAR	25	1.18	0.53–2.80	0.07–24.03	
	HS&DR	6	1.88	1.71–10.82	1.69–18.87	
	PHR	6	7.62	1.79–17.06	1.69–20.57	
Retention rate (percentage of randomised participants retained and assessed in primary outcome)	All	388	88%	80%–97%	0%–100%	
	HTA	303	89%	80%–97%	0%–100%	0.118*
	EME	39	89%	80%–97%	47%–100%	
	PGfAR	31	84%	78%–91%	43%–100%	
	HS&DR	9	82%	73%–89%	68%–99%	
	PHR	6	85%	78%–90%	74%–92%	

*P values are reported from a Kruskal-Wallis test.

EME, efficacy and mechanism evaluation; HS&DR, Health Services and Delivery Research; HTA, Health Technology Assessment; PGfAR, Programme Grants for Applied Research; PHR, Public Health Research.

The median consent rate (percentage of eligible participants consented and randomised) was found to be 72% (IQR: 50%–88%). Table 4 shows some variability in consent rates between the journals with the HS&DR journal having the largest median rate (81%, IQR: 60%–97%) and the PHR journal the lowest (57%, IQR: 40%–68%). However, there is not an overall statistically significant difference in consent rates between the five NIHR journals (p=0.225). The median retention rate (per cent of randomised participants retained and assessed in the analysis of the primary outcome) was found to be 88% (IQR: 80%–97%). There were four trials^25 26 28 39^ with a retention rate of 0%, these trials were all stopped early due to problems with recruitment and the planned statistical analysis for the primary outcome was not performed. Retention rates do not differ greatly between the five NIHR journals (p=0.118) (table 4).

The trial recruitment and retention rates are summarised by trial characteristics in tables 5 and 6, respectively. There is some statistical evidence of an association between the setting of the trial, final recruitment target and the total number of participants recruited but the median rates show no clear patterns to these associations.

**Table 5:** Association between recruitment rate (number of participants/centre/month) and trial characteristics

Characteristic (n=365)		n	Median	IQR	P value
Setting	Hospital	212	0.90	0.4–2.29	0.009*†
	General practice	51	0.71	0.32–1.18	
	Mixed	56	1.01	0.47–2.64	
	Community	29	2.44	0.62–6.41	
	Other	17	1.89	0.76–11.7	
Arms	2	278	1.10	0.41–2.76	0.935‡
	3	55	0.85	0.45–2.1	
	4	22	1.04	0.57–1.91	
	>4	10	0.85	0.42–8.85	
Control type	Placebo	85	0.84	0.38–1.93	0.145§
	Active	280	1.03	0.43–3.22	
Original target recruitment	≤200	41	1.18	0.47–2.65	0.008‡
	201–400	93	0.78	0.36–2.01	
	401–600	84	0.84	0.43–1.96	
	601–800	40	1.13	0.46–2.88	
	>800	105	1.49	0.55–4.72	
Final target recruitment	≤200	45	0.89	0.27–2.55	<0.001‡
	201–400	103	0.76	0.34–1.96	
	401–600	83	0.86	0.44–2.26	
	601–800	41	1.17	0.57–4.23	
	>800	93	1.66	0.58–5.17	
Total recruitment	≤200	63	0.50	0.17–1.6	<0.001‡
	201–400	90	0.78	0.37–2.07	
	401–600	81	1.15	0.49–2.41	
	601–800	39	1.03	0.57–3.85	
	>800	92	1.96	0.68–6.23	
Timing of final follow-up	≤1 month	19	1.29	0.42–2.26	0.054‡
	1< months ≤6	82	1.14	0.38–4.14	
	6< months ≤18	170	0.98	0.46–2.33	
	>18 months	85	0.71	0.36–2.02	

*The category ‘other’ was not included in Kruskal-Wallis test.

†P values are reported from a Kruskal-Wallis test.

‡P values are reported from a Jonckheere-Terpstra test.

§P values are reported from a Mann-Whitney U test.

**Table 6:** Association between the trial retention rate (% of randomised participants with valid primary outcome data for analysis) and trial characteristics

Characteristic (n=388)		n	Median	IQR	P value
Setting	Hospital	219	91.5	82.2–97.8	0.001*†
	General practice	55	84.0	76.6–91.3	
	Mixed	61	87.3	79.7–97.3	
	Community	34	84.9	75.4–90.8	
	Other	19	84.2	74.9–96.5	
Arms	2	290	89.9	81–97.4	<0.001‡
	3	61	84.4	72.4–93.6	
	4	24	83.2	79.6–88.2	
	>4	13	80.2	73.4–96.4	
Control type	Placebo	87	89.8	79.1–97.3	0.614§
	Active	301	87.8	80.3–96.4	
Final target recruitment	≤200	53	88.6	79.6–96.4	0.003‡
	201–400	112	86.1	77.1–94.1	
	401–600	84	86.8	78.9–95.7	
	601–800	42	84.4	80.4–90.9	
	>800	97	96.3	85.3–99.1	
Total recruitment	≤200	72	87.9	74.5–96.2	0.001‡
	201–400	99	87.3	79.3–94.9	
	401–600	82	86.4	80.6–94.1	
	601–800	39	86.2	82.2–91.4	
	>800	96	95.8	82.4–99	
Timing of final follow-up	≤1 month	20	92.2	78.7–99	0.518‡
	1< months ≤6	87	88.5	79.8–96.7	
	6< months ≤18	181	88.2	79.5–96.4	
	>18 months	88	87.8	80–95.5	

*The category ‘other’ was not included in Kruskal-Wallis test.

†P values are reported from a Kruskal-Wallis test.

‡P values are reported from a Jonckheere-Terpstra test.

§P values are reported from a Mann-Whitney U test.

The results of the current review, in terms of successful recruitment to target sample size, have been compared with three previous reviews^5 7 8^ in table 7. As this review updates the findings of Walters *et al*^8^ and due to there being some overlap with the trials included in Sully *et al*^5^; a column has been included for the non-overlapping time interval (2017–2020) in addition to the full time interval (1997–2020). Table 7 shows that 61% (107/174) of trials in the period 2017–2020 recruited 100% of the original target sample size which is higher than the previous periods/reviews. The target sample size was revised in 31% (54/174) of trials; and the revision was downwards for 57% (31/54) of trials. An extension, to the trial timelines, was reported in 37% (65/174) of trials and this was higher than the review by Walters *et al*^8^ (32% (49/151)) but lower than the reviews by McDonald *et al*^7^ (54% (65/122)) and Sully *et al*^5^ (45% (33/73)).

**Table 7:** Comparison of the current review with three previous reviews in terms of successful recruitment to target sample size and extensions to recruitment

Review	McDonald *et al*^7^	Sully *et al*^5^	Walters *et al*^8^	This study	This study
Recruitment period	1994–2002	2002–2008	2004–2016	2017–2020	1997–2020
No of trials in the study	N=122	N=73	N=151	N=174	N=388
Recruited 100% of original target	38 of 122 (31%)	40 of 73 (55%)	61 of 151 (40%)	107 of 174 (61%)	207 of 388 (53%)
Original target was revised	42 of 122 (34%)	14 of 73 (19%)	52 of 151 (34%)	54 of 174 (31%)	118 of 388 (30%)
Original target revised upward	6 of 42 (14%)	5 of 14 (36%)	11 of 52 (21%)	23 of 54 (43%)	39 of 118 (33%)
Original target revised downward	36 of 42 (86%)	9 of 14 (64%)	41 of 52 (79%)	31 of 54 (57%)	79 of 118 (67%)
Recruited 80% of original target	67 of 122 (55%)	57 of 73 (78%)	95 of 151 (63%)	139 of 174 (80%)	288 of 388 (74%)
Recruited 100% of revised target	19 of 42 (45%)	10 of 14 (71%)	28 of 52 (54%)	35 of 54 (65%)	80 of 118 (68%)
Recruited 80% of revised target	34 of 42 (80%)	13 of 14 (93%)	48 of 52 (92%)	48 of 54 (89%)	107 of 118 (91%)
Extended their recruitment	65 of 122 (54%)	33 of 73 (45%)	49 of 151 (32%)	65 of 174 (37%)	128 of 388 (33%)

**Figure 3:**
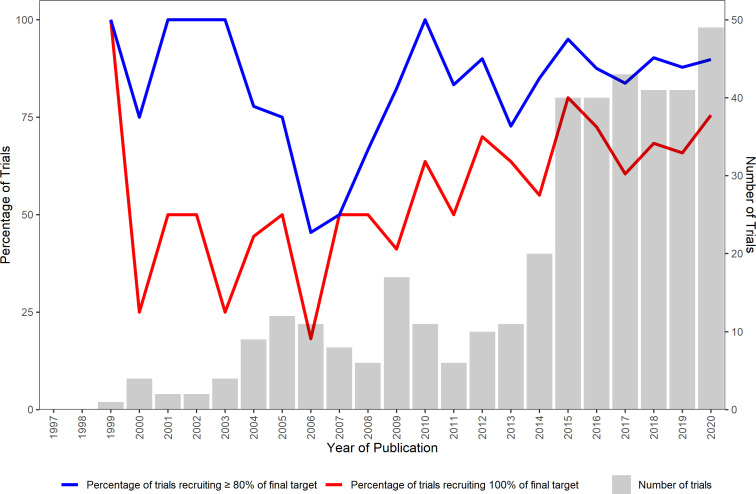
Number of trials and percentage of trials recruiting 100% and ≥80% of the final sample size target from 1997 to 2020

Figure 3 shows the percentage of trials recruiting 100% of the final target and 80% or more of the final target by publication year. There is no clear trend in the percentage of trials recruiting 100% of the final target for the earlier years (1999–2006) but there is evidence of an upward trend for the years 2007 to 2020.

## Discussion and conclusions

This study has systematically conducted a review of the recruitment and retention data from a cohort of 388 trials published in the NIHR Journals Library between 1997 and 2020. This review found that the final target sample size was achieved in 63% (245/388) of RCTs; the median recruitment rate was 0.95 (IQR: 0.42–2.60) participants per centre per month; the median consent rate was 72% (IQR: 50%–88%); and the median retention rate was 88% (IQR: 80%–97%).

This review found that 53% (207/388) of publicly funded RCTs achieved their original target sample size. Restricting the time period to 2017–2020 the figure is 61% (107/174), this is higher than the previous figures of 55% and 40% found in the reviews by Sully *et al*^5^ and Walters *et al*.^8^ This is also reflected in the percentage of trials recruiting to 100% of their final target where there is some evidence of an upward trend for the years 2007 to 2020. However, there is some evidence of a difference in recruitment rates between the five NIHR journals, and therefore, any improvement may be due to the inclusion of trials from the journals (EME, PGfAR, HS&DR and PHR) that were not included in the review by Walters *et al*.^8^ There is still cause for some concern with 30% (118/388) of trials revising their original recruitment target with the majority (67% (79/118)) revising the target downwards, and a third (128/388) of trials having an extension to their recruitment period. These findings remain consistent with the concerns expressed by CTU directors.^13^

The median consent and retention rate are consistent with the result of Walters *et al*.^8^ The retention figure may be an overestimate as it will be affected by trials using time to event outcomes, where missing outcomes are censored at the time of loss to follow-up but included in analyses using survival models. The target sample size for any trial should allow for participant withdrawals and loss to follow-up^40^ with the expected withdrawal proportion obtained from reports of studies conducted in the same clinical area.^20^ However, if no such information is available then a pragmatic approach would be to use the median retention rate from this review (88%) and assume an expected withdrawal proportion of at least 10%.

This study has the following limitations. First, the review was restricted to publicly funded trials published in the NIHR Journals Library, which may limit the generalisability of the findings. It is possible that that problems with recruitment and retention of participants in NHIR funded trials will be less pronounced than in other trials due to the rigorous appraisal of feasibility prior to funding and the ongoing monitoring during the conduct of the trial. However, as the NIHR Journals Library intends to publish all research from EME, HS&DR, HTA, PGfAR and PHR funded projects, it has less chance of publication bias compared with a review of other journals where publishing is more selective and information related to recruitment is published in less detail. Second, the data extraction was conducted by several independent reviewers and although reviewers conferred to try and ensure consistency and quality assurance checks were completed on a sample of reports, it is possible that errors have occurred. Third, the calculation of recruitment rates was limited by the information reported. For some trials, centre-specific recruitment information was not available meaning that crude recruitment rates, assuming all centres were recruiting for the same time period, were calculated. In these cases, the calculated recruitment rate may be an underestimate of the true recruitment rate.

This review found considerable variation in the consent, recruitment and retention rates in publicly funded RCTs. Although the majority of (6 out of 10) trials in this review achieved their final target sample; 3 out of 10 trials published in NIHR Journals Library revised their original target sample size (downwards in 7 out of 10 trials). Investigators should bear this in mind at the planning stage of their study and not be overly optimistic about their recruitment projections.

## Supplementary Material

Reviewer comments

Author's manuscript

## Data Availability

Data are available on reasonable request. The information extracted in this review is based on published trials in the NIHR Journals Library. The extracted data is available on reasonable request from the corresponding author at r.jacques@sheffield.ac.uk.
